# LncRNA KASRT Serves as a Potential Treatment Target by Regulating SRSF1-Related KLF6 Alternative Splicing and the P21/CCND1 Pathway in Osteosarcoma: An *In Vitro* and *In Vivo* Study

**DOI:** 10.3389/fonc.2021.700963

**Published:** 2021-09-09

**Authors:** Kai Chen, Cheng Li, Shuai Huang, Yu Chen, Xiaodong Zhu

**Affiliations:** ^1^Department of Orthopedics, Changhai Hospital, Second Military Medical University, Shanghai, China; ^2^Department of Orthopedics, Huashan Hospital, Fudan University, Shanghai, China; ^3^Department of Spine Surgery, Shanghai Renji Hospital, Shanghai JiaoTong University School of Medicine, Shanghai, China

**Keywords:** osteosarcoma, lncRNA KASRT, SRSF1, KLF6 alternative splicing, malignant behaviors

## Abstract

**Purpose:**

Long non-coding RNA KLF6 alternative splicing regulating transcript (lnc-KASRT) locates within the intronic region of SRSF1, possessing the potential to regulate KLF6 alternative splicing to promote carcinogenicity. Then, the current *in vitro* and *in vivo* study aimed to investigate the effect of lnc-KASRT on regulating tumor malignant behaviors, and the implication of its interaction with KLF6 alternative splicing in osteosarcoma.

**Methods:**

Lnc-KASRT overexpression or knockdown plasmid was transfected into U-2OS and Saos-2 cells. Then, KLF6-SV1 knockdown plasmid with or without lnc-KASRT overexpression plasmid was transfected into these cells for compensative experiments. *In vivo*, lnc-KASRT overexpression or knockdown Saos-2 cells were injected in mice for tumor xenograft construction.

**Results:**

Lnc-KASRT expression was increased in most osteosarcoma cell lines compared to control cell line. Lnc-KASRT overexpression promoted cell viability, mobility, and anti-apoptotic marker expression, while reducing apoptosis rate and pro-apoptotic marker expression; meanwhile, it regulated SRSF1, KLF6 alternative splicing (increased KLF6-splice variant 1 (KLF6-SV1), decreased KLF6-wild type (KLF6-WT)), and followed P21/CCND1 pathway in U-2OS/Saos-2 cells. The lnc-KASRT knockdown exhibited opposite trends. Subsequent compensative experiments disclosed that KLF6-SV1 knockdown attenuated most of the tumor-promoting effects of lnc-KASRT overexpression in U-2OS/Saos-2 cells. *In vivo* experiments further validated that lnc-KASRT enhanced tumor growth and reduced tumor apoptosis; meanwhile, it also increased tumor KLF6-SV1, MMP-1, and MMP-9 expressions but decreased tumor SRSF1 and KLF6-WT expressions in xenograft mice.

**Conclusion:**

Lnc-KASRT serves as a potential treatment target *via* regulating SRSF1-related KLF6 alternative splicing and following P21/CCND1 pathway in osteosarcoma.

## Introduction

Osteosarcoma is one the most vicious cancers that primarily affect children and young adults, with a low overall incidence rate of 3–5 per million in males and 2–4 per million in females each year (1, 2). Due to the exasperated features of osteosarcoma, such as high heterogeneity, rapid growth rate, and strong invasive ability, a nonnegligible proportion of patients are diagnosed with distant metastases, and its prognosis is very dismal (3–5). To improve the survival of patients with osteosarcoma, continuous and sincere efforts have been made to enhance surgical technology, neoadjuvant/adjuvant treatment strategies, novel targeted drugs, and personalized medicine, and these approaches have progressed to some extent (6–9). However, the prognosis of osteosarcoma patients is still far from satisfactory. Therefore, it is crucial to explore the underlying mechanism of osteosarcoma development and progression to identify potentially useful treatment targets.

Krüppel-like factor 6 (KLF6) is a key transcription factor that regulates various pivotal biological processes, such as proliferation, differentiation, metabolism, and inflammation (10). Due to the functions mentioned above, accumulating studies have reported that KLF6 is deeply involved in tumor pathogenesis, including osteosarcoma. For instance, a study revealed that KLF6 represses cell proliferation and invasion while promoting cell apoptosis in a p21-dependent manner in osteosarcoma; furthermore, another study demonstrated that KLF6 is insufficiently expressed in osteosarcoma cells and tissues (11–13). Interestingly, further studies have revealed that the KLF6 gene encodes KLF6 splice variant 1 (KLF6-SV1) *via* alternative splicing, which could indirectly promote carcinogenesis by binding to KLF6 wild type (KLF6-WT) and proteases involved in degradation, as well as by directly acting as an oncogene through its regulation of NOXA, TWIST genes, and the PI3K/AKT pathway (14–17). As the regulator of KLF6 alternative splicing, serine and arginine rich splicing factor 1 (SRSF1) plays a crucial role (18, 19). In the SRSF1 intronic region, there exists an ultra-conserved region (UCR) 419 (UCR-419), which is 280 bp in length and lacks a protein-coding ability (https://www.ncbi.nlm.nih.gov/nuccore/NC_000017.11, access number: NC_000017.11 (SRSF1)). In our preliminary experiments, UCR-419 promoted KLF6-LV1 coding but weakened KLF6-WT coding by directly sponging SRSF1. Since this property of UCR-419 fit the definition of a long noncoding RNA (lncRNA), we named it lncRNA KLF6 Alternative Splicing Regulating Transcript (KASRT) (lnc-KASRT) (20).

Then, our current *in vitro* and *in vivo* study aimed to investigate the effect of lnc-KASRT on regulating malignant tumor behaviors and the implication of its interaction with KLF6 alternative splicing in osteosarcoma.

## Methods

### Cell Culture

Human osteosarcoma cell lines, including U-2OS, Saos-2, MG-63, MNNHG/HOS, and human osteoblast hFOB1.19, were purchased from American Type Culture Collection (ATCC) (VA, USA) and cultured according to the instructions from the supplier (www.atcc.org). U-2OS and Saos-2 cells were cultured in 90% McCoy’s 5A (modified) medium (Gibco, USA) supplemented with 10% fetal bovine serum (FBS) (Gibco, USA). MG-63 and MNNG/HOS cells were grown in 90% Minimum Essential Medium α (MEM α) (Gibco, USA) supplemented with 10% FBS (Gibco, USA). hFOB1.19 cells were incubated in 90% Dulbecco’s modified Eagle’s medium/nutrient mixture F-12 (DMEM/F12) supplemented with 10% FBS (Gibco, USA). All cells were maintained at 37°C in a humidified atmosphere of 95% air and 5% CO_2_. After being cultured, reverse transcription-quantitative polymerase chain reaction (RT-qPCR) assays were performed to determine Lnc-KASRT expression in the hFOB1.19, U-2OS, Saos-2, MG-63, and MNNHG/HOS cell lines, with hFOB1.19 serving as a control.

### Lnc-KASRT Plasmid Transfection

Overexpression plasmids, including the Lnc-KASRT overexpression plasmid and control overexpression plasmid, were constructed with pEX-2 by Guangzhou RiboBio Co., Ltd. (Guangzhou, China). Knockdown plasmids, including the Lnc-KASRT knockdown plasmid and control knockdown plasmid, were constructed with pRNAT-U6.1/Neo by Guangzhou RiboBio Co., Ltd. (Guangzhou, China). The constructed plasmids were transfected into U-2OS cells and Saos-2 cells using Lipofectamine 2000 (Invitrogen, USA) according to the manufacturer’s instructions and a previously reported method (21). Following transfection, the cells in each cell line (U-2OS and Saos-2) were divided into four groups: Lnc-KASRT overexpression cells (KASRT(+) group); control overexpression cells (NC(+) group); Lnc-KASRT knockdown cells (KASRT(-) group); and control knockdown cells (NC(-) group). In the above groups, the determination of Lnc-KASRT expression was performed by RT-qPCR assays at 24 h post transfection. The cell viability assessment was carried out with the use of Cell Counting Kit-8 (CCK-8) (Dojindo, Japan) at 0, 24, 48, and 72 h. Cell apoptosis was measured by Annexin V/propidium iodide (AV/PI) assay with an Annexin V-FITC apoptosis detection kit (R&D, USA) at 48 h. The CCK-8 assay and AV/PI assay were completed following the manufacturer’s instructions. The assessment of migration ability and invasion ability was performed at 48 h through wound healing assays and Transwell assays with Matrigel basement membrane matrix (BD, USA)-coated chambers (Corning, USA), and all procedures were performed according to the methods described in previous studies (22, 23). Moreover, the expression of serine- and arginine-rich splicing factor 1 (SRSF1), Kruppel-like factor 6 (KLF-6) transcript variant D (also known as KLF-6-SV1), and KLF-6 wild type (KLF-6-WT) was detected by RT-qPCR and Western blot analysis at 48 h. Notably, in U-2OS cells after transfection, chemosensitivity to cisplatin, methotrexate, and doxorubicin was detected by coculture with 0.0–6.4 μM cisplatin, 0.0–6.4 μM methotrexate, and 0–32 nM doxorubicin.

### KLF-6-SV1 Plasmid Transfection

The KLF-6-SV1 knockdown plasmid and control knockdown plasmid were constructed by Guangzhou RiboBio Co., Ltd. (Guangzhou, China) using pRNAT-U6.1/Neo. KLF-6-SV1 knockdown plasmid and control knockdown plasmid were transfected into NC(+) group cells and KASRT(+) group cells using Lipofectamine 2000 (Invitrogen, USA) according to the manufacturer’s instructions and to the method previously reported (21), which generated another four groups: NC(+)&KLF-6-SV1(-) group, NC(+)&NC(-) group, KASRT(+)&KLF-6-SV1(-) group, and KASRT(+)&NC(-) group. KLF-6-SV1 expression was detected by performing RT-qPCR and Western blot experiments at 24 h post transfection, and Lnc-KASRT expression, cell viability, cell apoptosis, migration ability, and invasion ability were determined according to the methods described above. Additionally, the expression of SRSF1, P21, and Cyclin D1 (CCND1) was detected by RT-qPCR and Western blot assays at 48 h post transfection.

### Stable Infection Cell Construction

The pLV-CMV-IRES-Puro vector (Hanbio, China) was used to construct the Lnc-KASRT overexpression plasmid, and the pLV-U6-RFP-T2A-Puro vector (Hanbio, China) was used to construct the Lnc-KASRT knockdown plasmid according to the methods described previously (24). To generate overexpression and knockdown lentiviruses, plasmid and envelope helper vectors were cotransfected into 293T cells using Lipofectamine 2000 (Invitrogen, USA). Control lentivirus was provided by Hanbio Biotechnology Co., Ltd. (Shanghai, China). After harvesting, the constructed lentivirus was used to infect Saos-2 cells, and then the stably infected Saos-2 cells were screened with 2 μg/mL puromycin (Sigma, USA). These cells were marked as KASRT(+) cells, KASRT(-) cells, and NC cells depending on the infected lentivirus (Lnc-KASRT overexpression lentivirus, Lnc-KASRT knockdown lentivirus, or control lentivirus).

### Tumor Xenograft Model Construction

All animal experiments were approved by the Institutional Animal Care and Use Committees (IACUC) of our institution and were performed in accordance with institutional guidelines and ethical standards. A tumor xenograft model was constructed according to methods described previously (25). Male BALB/C nude mice (5–6 weeks of age, 16–18 g) were maintained under specific pathogen-free conditions. Approximately 2 × 10^6^ KASRT(+) cells, KASRT(-), cells and NC cells were suspended in 100 μl PBS and then injected subcutaneously into the right side of the posterior flank of nude mice. The mice were divided into the KASRT(+) group, KASRT(-) group, and NC group. Tumor volumes were examined every 7 days using a Vernier caliper (Mitutoyo, Japan). Tumor volumes were calculated using the equation: Volume = a*b^2^/2 (mm^3^), where a is the largest diameter and b is the perpendicular diameter. Four weeks after cell injection, mice were sacrificed, and tumors were harvested. After weighing, tumors were stored properly for further analysis. For tumors placed in 10% formalin and embedded in paraffin, hematoxylin-eosin (HE) staining was performed for histological analysis, terminal deoxynucleotidyl transferase (TdT)-mediated dUTP nick end labeling (TUNEL) assay was carried out to detect cell apoptosis, and immunochemistry (IHC) was performed to detect protein expression of SRSF1, KLF-6-SV1, KLF-6-WT, MMP-1, and MMP-9. For tumors stored at -80°C, SRSF1, KLF-6-SV1, KLF-6-WT, MMP-1, and MMP-9 mRNA expression was evaluated by RT-qPCR assays.

### RT-qPCR

Total RNA was extracted by TRIzol™ Reagent (Invitrogen, USA), and then reverse transcription and PCR were performed with the PrimeScript™ RT reagent Kit (Takara, Japan) and TB Green™ Fast qPCR Mix (Takara, Japan), respectively. Gene expression was calculated by the 2^-△△Ct^ method with GAPDH as the internal reference according to a previously reported method (26). Primer sequences are listed in **Supplementary Table 1**.

### Western Blot

Western blotting was performed by standard procedures according to previous studies (22, 23). Protein extraction and quantification were performed using RIPA buffer (Sigma, USA), and a bicinchoninic acid kit was used for protein determination (Sigma, USA). Furthermore, NuPAGE™ 4–20% Tris-Acetate Midi Protein Gels (Thermo, USA), XCell SureLock™ Mini-Cell (Invitrogen, USA), Pierce™ECL Plus Western Blotting Substrate (Invitrogen, USA), X-ray film (Kodak, USA), and Gel Imager (Thermo, USA) were also used. Antibody information is presented in **Supplementary Table 2**.

### HE Staining, TUNEL Assay, and IHC

HE staining, TUNEL assay, and IHC experiments were performed according to the methods described previously (26, 27). The formalin-fixed and paraffin-embedded tumor specimens were sliced into 4 μm sections. Then, a hematoxylin and eosin staining kit (Beyotime, China) was used for HE staining, which was performed in accordance with the kit’s standard protocol. The TUNEL assay was performed using a TumorTACS *in situ* apoptosis kit (R&D, USA) according to the manufacturer’s manual. Furthermore, IHC was completed according to the standard operating protocol of The Early Detection Research Network (EDRN), and the antibodies used for the IHC assays are listed in **Supplementary Table 2**. Images were obtained using an Olympus BX41 microscope (Olympus, Japan).

### Statistical Analysis

Bar charts and line charts with error bars were used to display a statistical summary of the mean value and standard deviation (SD). Comparison between two independent samples was determined by the unpaired t test; multiple comparisons between a control group and other experimental groups were determined by one-way ANOVA followed by Dunnett’s test; multiple comparisons between each group were determined by one-way ANOVA followed by Tukey’s test. Statistical analysis and graph plotting were performed using GraphPad Prism 7.02 software (GraphPad Software Inc., USA). Significance was defined as a *P* < 0.05, which is displayed as *P < 0.05; **P < 0.01; ***P < 0.001, and nonsignificant is marked as NS.

## Results

### Lnc-KASRT Promoted Osteosarcoma Cell Viability and Mobility

**Figure 1:**
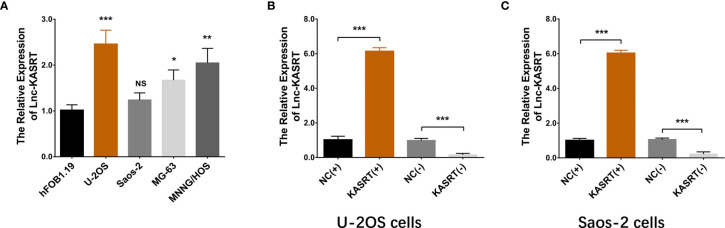
Lnc-KASRT expression. Lnc-KASRT expression among osteosarcoma cell lines and a control cell line **(A)**; Lnc-KASRT expression after transfection in both U-2OS **(B)** and Saos-2 cells **(C)**. *P < 0.05, **P < 0.01, ***P < 0.001, NS, Not significance (P > 0.05).

**Figure 2:**
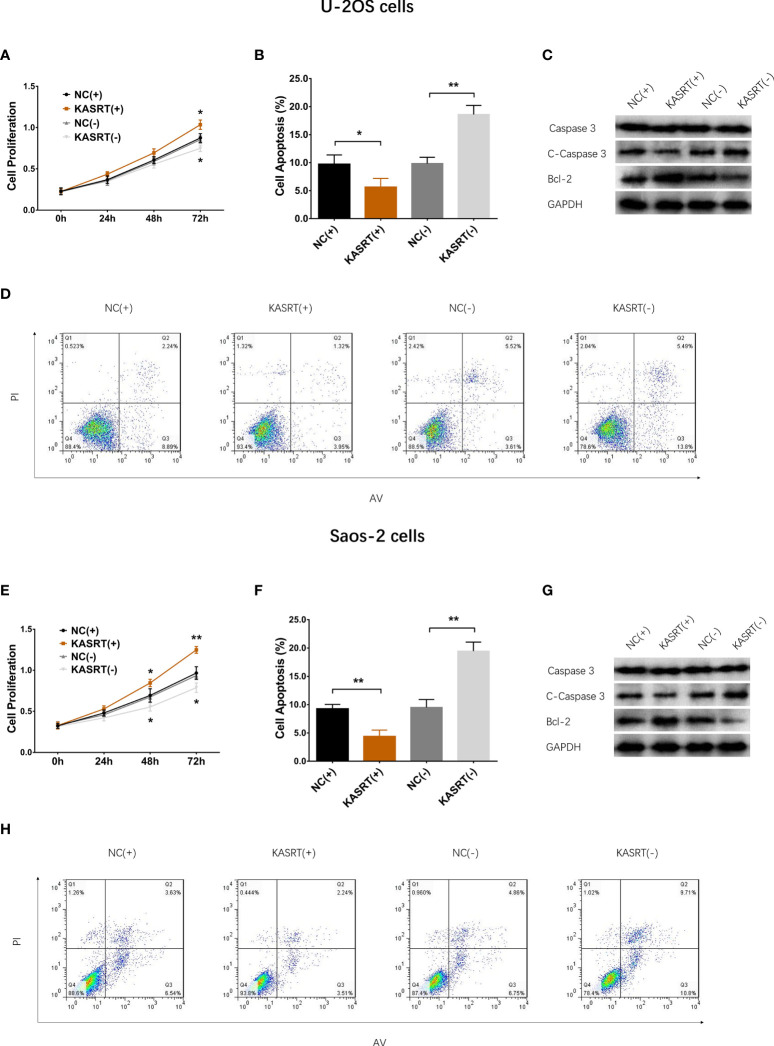
Cell proliferation and apoptosis. Cell proliferation **(A)**, cell apoptosis rate **(B)**, pro-/anti-apoptotic marker expression **(C)**, and AV/PI images **(D)** among the groups of U-2OS cells. Cell proliferation **(E)**, cell apoptosis rate **(F)**, pro-/anti-apoptotic marker expression **(G)**, and AV/PI images **(H)** among the groups of Saos-2 cells. *P < 0.05, **P < 0.01.

**Figure 3:**
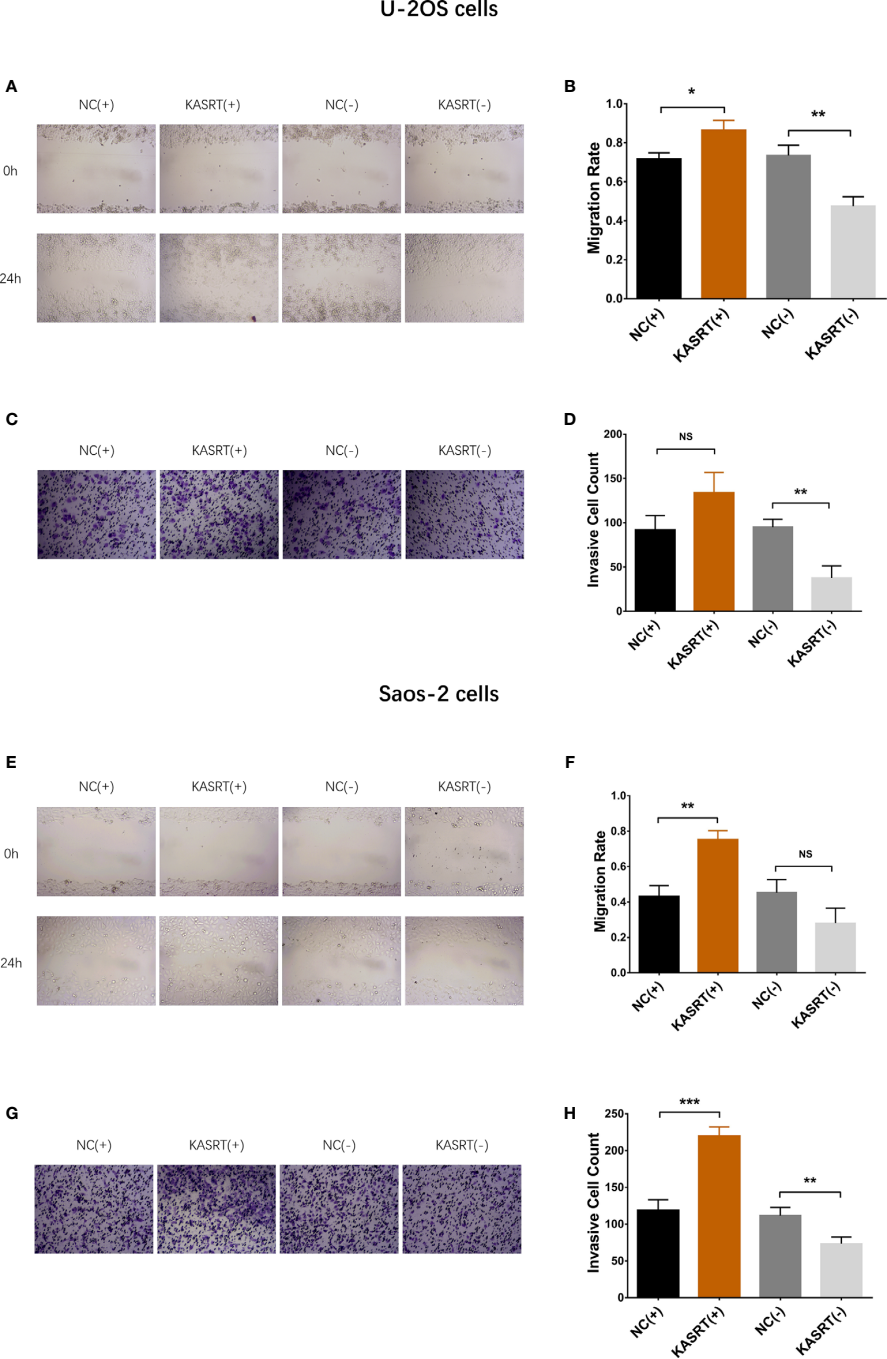
Cell migration and invasion. Cell migration **(A, B)** and invasion **(C, D)** among groups of U-2OS cells. Cell migration **(E, F)** and invasion **(G, H)** among the groups of Saos-2 cells. *P < 0.05, **P < 0.01, ***P < 0.001, NS, Not significance (P > 0.05).

Lnc-KASRT was elevated in several osteosarcoma cell lines, such as U-2OS, MG-63, and MNNG/HOS, but was not changed in the Saos-2 cell line compared to the control cell line hFOB1.19 (**Figure 1A**). After transfection in both U-2OS and Saos-2 cells, lnc-KASRT was increased in the KASRT (+) group compared to the NC (+) group but was decreased in the KASRT (-) group compared to the NC (-) group, indicating successful transfection (**Figures 1B, C**). Regarding cell viability, lnc-KASRT overexpression promoted cell proliferation and expression of the antiapoptotic marker BCL-2 and reduced the cell apoptosis rate and expression of the proapoptotic marker C-Caspase3 in both U-2OS (**Figures 2A–D**) and Saos-2 cells (**Figures 2E–H**). In terms of cell mobility, lnc-KASRT overexpression enhanced cell migration and invasion in both U-2OS (**Figures 3A–D**) and Saos-2 cells (**Figures 3E–H**). Moreover, lnc-KASRT knockdown exhibited the opposite effect on these cell functions (**Figures 2A–H**) (**Figures 3A–H**). However, lnc-KASRT had little effect on chemosensitivity in U-2OS cells, except that it had a slight effect on doxorubicin chemosensitivity in U-2OS cells (**Supplementary Figures S1A–C**); therefore, subsequent exploration of chemotherapy sensitivity was not continued.

### Lnc-KASRT Regulated KLF6 Alternative Splicing

**Figure 4:**
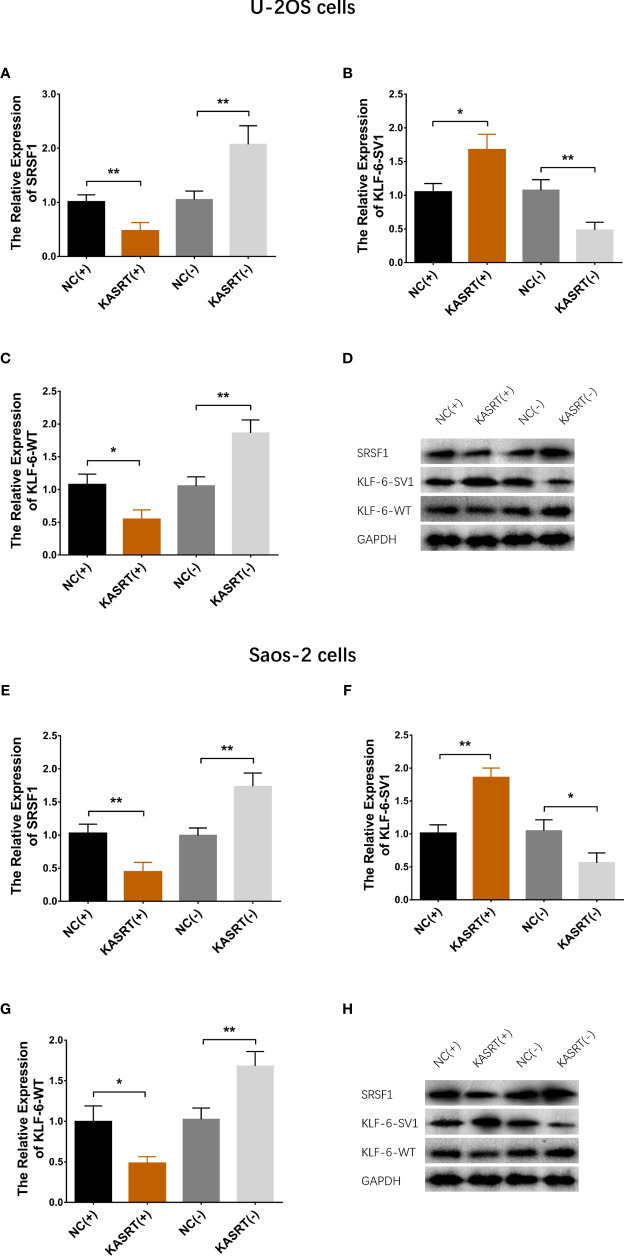
KLF6 alternative splicing. mRNA and protein expression of SRSF1, KLF6-SV1, and KLF6-WT in U-2OS **(A–D)** and Saos-2 cells **(E–H)**. *P < 0.05, **P < 0.01.

Lnc-KASRT overexpression upregulated KLF6-SV1 expression but downregulated SRSF1 and KLF6-WT expression in both U-2OS (**Figures 4A–D**) and Saos-2 cells (**Figures 4E–H**). At the same time, lnc-KASRT knockdown decreased KLF6-SV1 expression and increased SRSF1 and KLF6-WT expression in both U-2OS (**Figures 4A–D**) and Saos-2 cells (**Figures 4E–H**).

### KLF6-SV1 Knockdown Attenuated the Effect of lnc-KASRT on Osteosarcoma Cell Viability and Mobility

**Figure 5:**
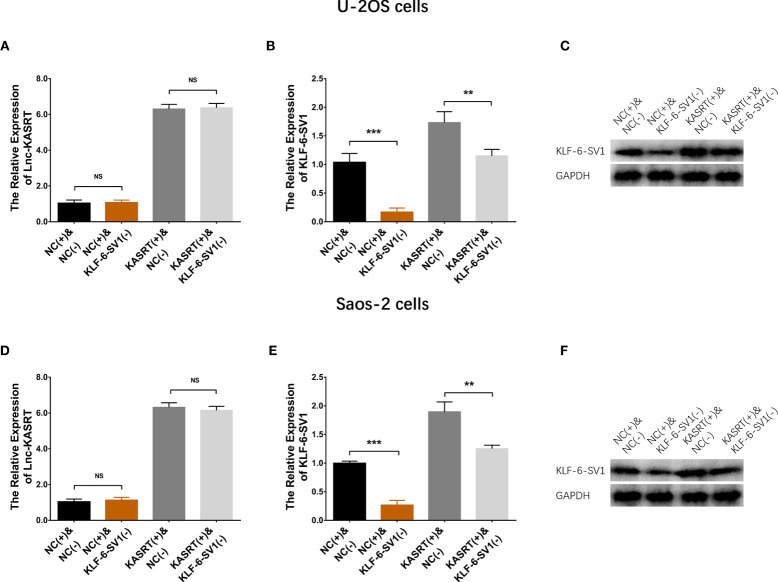
Lnc-KASRT and KLF6-SV1 expression in compensatory experiments. Lnc-KASRT **(A)**, KLF6-SV1 mRNA **(B)**, and protein **(C)** expression among the groups of U-2OS cells. Lnc-KASRT **(D)**, KLF6-SV1 mRNA **(E)**, and protein **(F)** expression among the groups of Saos-2 cells. **P < 0.01, **P < 0.001, NS, Not significance (P > 0.05).

**Figure 6:**
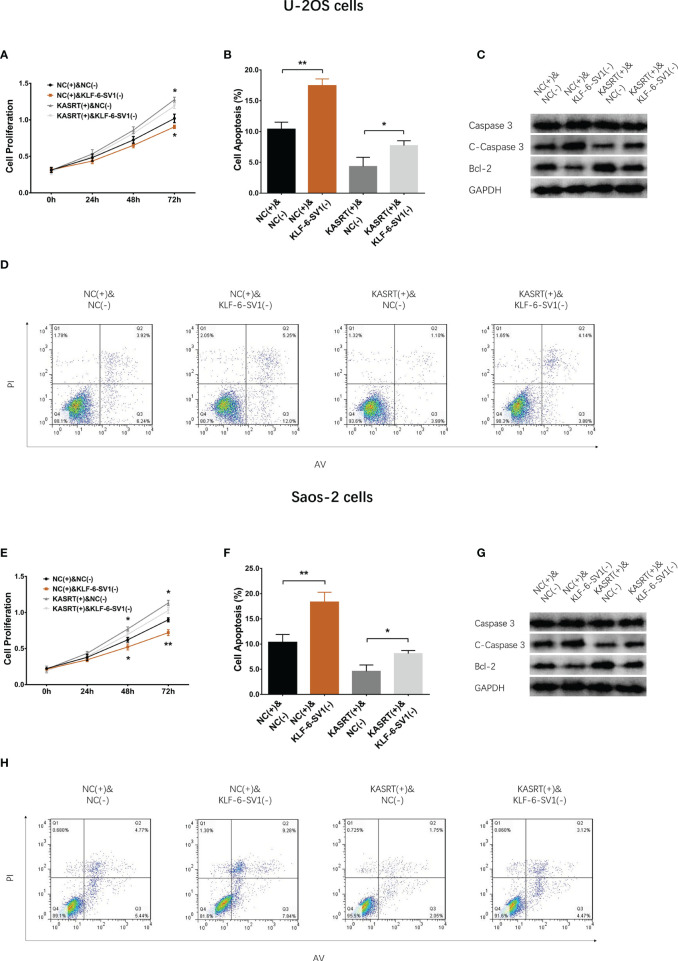
Cell proliferation and apoptosis in compensatory experiments. In compensatory experiments, cell proliferation **(A)**, cell apoptosis rate **(B)**, pro-/anti-apoptotic marker expression **(C)**, and AV/PI images **(D)** among groups in U-2OS cells; cell proliferation **(E)**, cell apoptosis rate **(F)**, pro-/anti-apoptotic marker expression **(G)**, and AV/PI images **(H)** among groups in Saos-2 cells. *P < 0.05, **P < 0.01.

**Figure 7:**
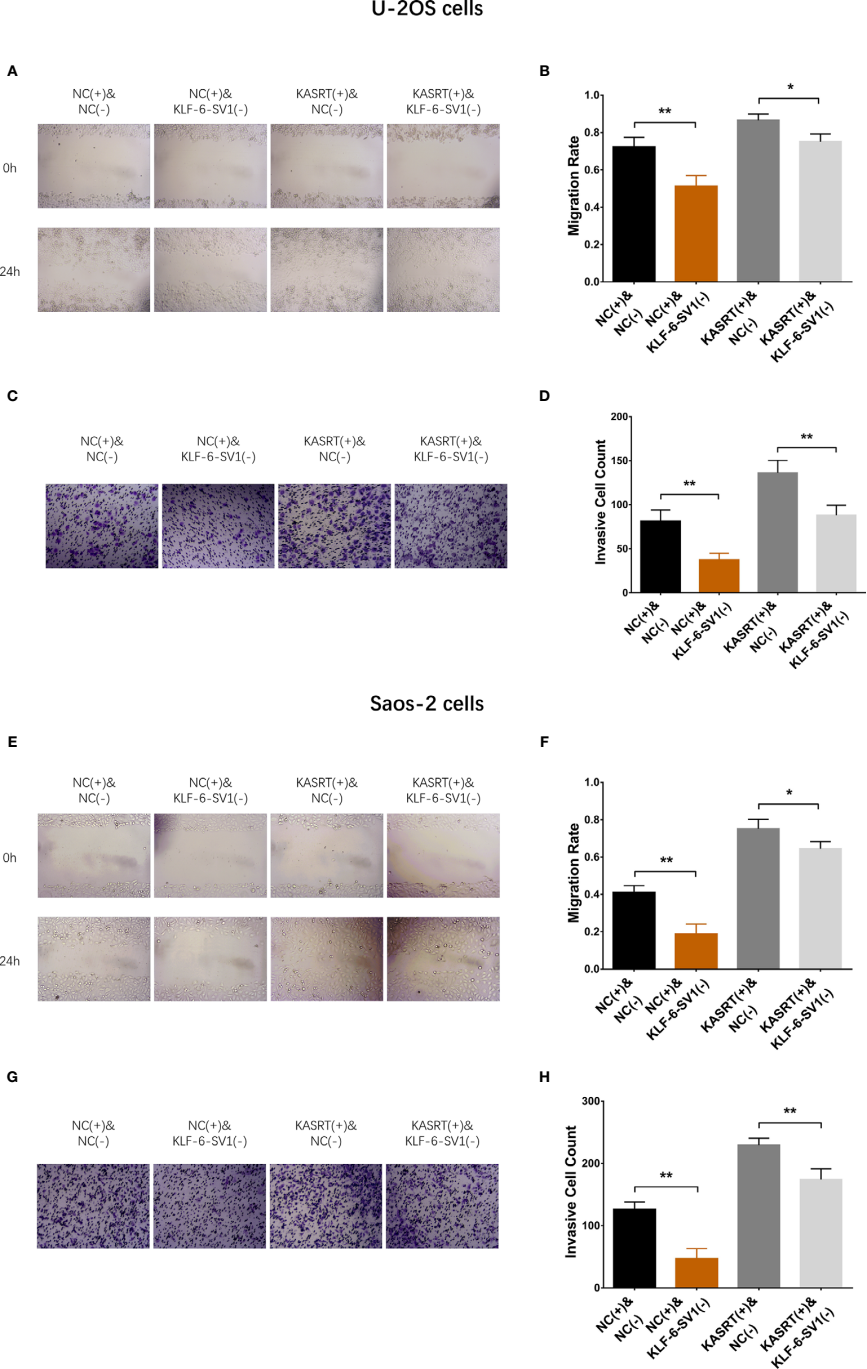
Cell migration and invasion in compensatory experiments. In compensatory experiments, cell migration **(A, B)** and invasion **(C, D)** among groups in U-2OS cells and cell migration **(E, F)** and invasion **(G, H)** among the groups of Saos-2 cells. *P < 0.05, **P < 0.01.

After transfection with its overexpression plasmid, lnc-KASRT was upregulated, while KLF6-SV1 was downregulated by its knockdown plasmid, indicating successful transfection. Further analyses revealed that lnc-KASRT positively regulated KLF6-SV1, but KLF6-SV1 did not affect lnc-KASRT in either U-2OS (**Figures 5A–C**) or Saos-2 cells (**Figures 5D–F**). Interestingly, KLF6-SV1 knockdown enhanced cell apoptosis while reducing cell proliferation, migration, and invasion, as reflected by AV/PI, CCK-8, wound healing, Transwell assays, and pro/anti-apoptotic marker expression in both U-2OS and Saos-2 cells (**Figures 6A–H**, **7A–H**). Importantly, further analyses showed that KLF6-SV1 knockdown impaired the effect of lnc-KASRT on regulating cell apoptosis, proliferation, migration, and invasion in both U-2OS and Saos-2 cells (**Figures 6A–H**, **7A–H**).

### Lnc-KASRT Regulated P21 and CCND1 *via* KLF6-SV1

**Figure 8:**
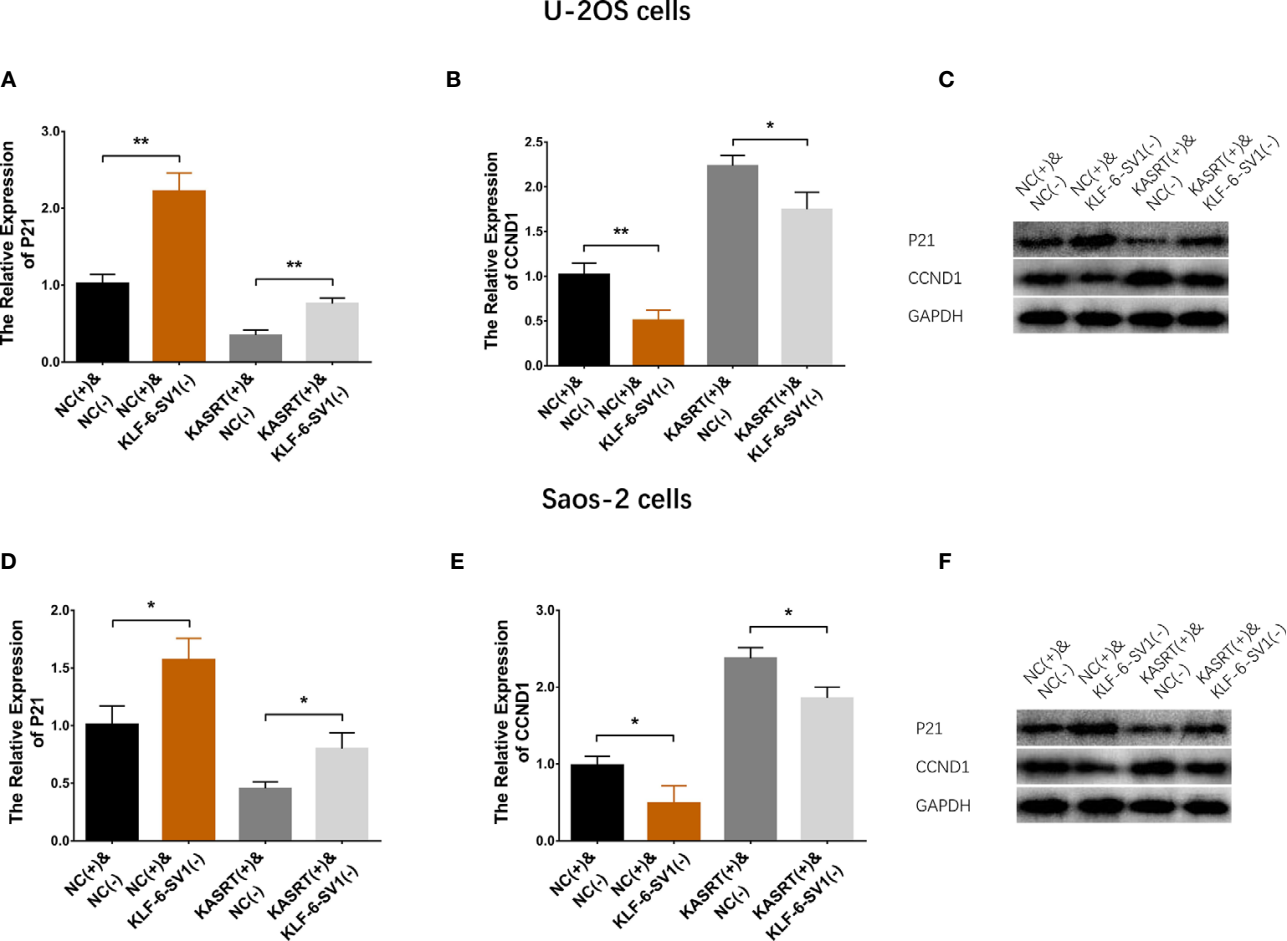
P21/CCND1 pathway in compensatory experiments. In compensatory experiments, P21 and CCND1 mRNA and protein expression among the groups of U-2OS cells **(A–C)** and Saos-2 cells **(D–F)**. *P < 0.05, **P < 0.01.

Lnc-KASRT overexpression downregulated P21 but upregulated CCND1; KLF6-SV1 knockdown increased P21 while reducing CCND1 in both U-2OS (**Figures 8A–C**) and Saos-2 cells (**Figures 8D–F**). Notably, KLF6-SV1 knockdown weakened the effect of lnc-KASRT regulation of P21 and CCND1 in both U-2OS and Saos-2 cells (**Figures 8A–F**).

### Lnc-KASRT Promoted Osteosarcoma Progression and KLF6 Alternative Splicing *In Vivo*

**Figure 9:**
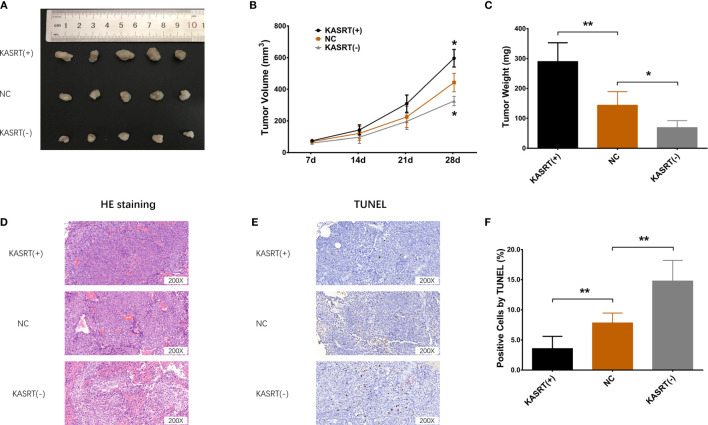
Osteosarcoma tumor growth and apoptosis. Tumor volume **(A, B)**, tumor weight **(C)**, HE staining **(D)**, and TUNEL staining **(E, F)** among the cell groups. *P < 0.05, **P < 0.01.

**Figure 10:**
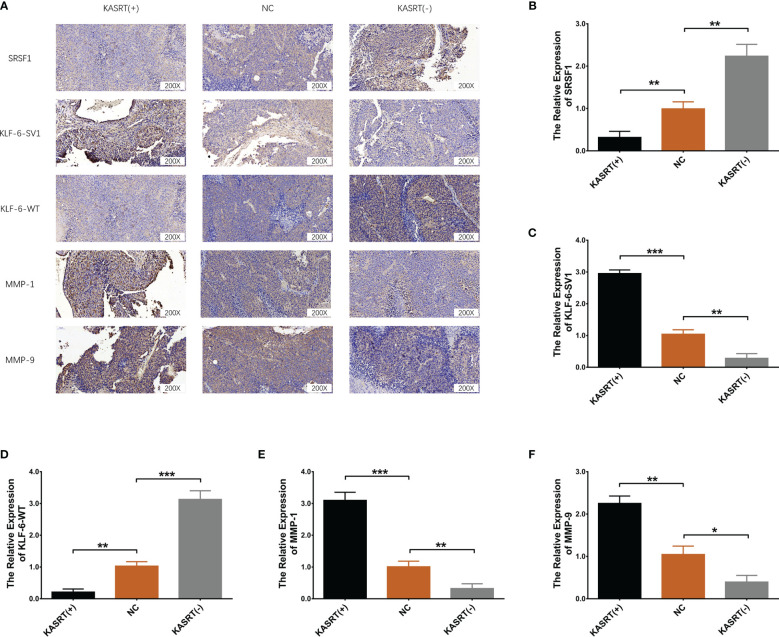
SRSF1, KLF6-SV1, KLF6-WT, MMP1, and MMP9 expression in osteosarcoma tissue. Protein expression of SRSF1, KLF6-SV1, KLF6-WT, MMP1, and MMP9 in osteosarcoma tissue **(A)**. mRNA expression of SRSF1 **(B)**, KLF6-SV1 **(C)**, KLF6-WT **(D)**, MMP1 **(E)**, and MMP9 **(F)** in osteosarcoma tissue. *P < 0.05, **P < 0.01, ***P < 0.001.

Lnc-KASRT overexpression facilitated tumor growth (**Figures 9A–D**) and reduced tumor apoptosis (**Figures 9E, F**) in osteosarcoma mice. In contrast, Lnc-KASRT knockdown repressed tumor growth while inducing HE-reflected necrosis and TUNEL-reflected tumor apoptosis in osteosarcoma mice (**Figures 9A–F**). Furthermore, lnc-KASRT overexpression increased KLF6-SV1, MMP1, and MMP9 expression and decreased SRSF1 and KLF6-WT expression in osteosarcoma mice (**Figures 10A–F**), but lnc-KASRT downregulation showed the opposite trend (**Figures 10A–F**).

## Discussion

Two decades ago, the alternative splicing ability of SRSF1 was revealed, and it has been shown to regulate many complicated biological processes comprising some important aspects of mRNA metabolism (such as mRNA splicing, stability, translation, etc.) and other mRNA-independent pathways (such as miRNA processing, protein sumoylation, nucleolar stress response, etc.) (28, 29). On account of the above features, SRSF1 is also considered a key carcinogenic factor that functions *via* multiple mechanisms, such as regulating extrinsic (for instance, Fas, Caspase-8, Caspase-2, c-FLIP) and intrinsic (Apaf-1, Caspase-9, ICAD) factors, genes encoding Bcl-2 proteins, IAPs, and p53 tumor suppressors (29, 30).

Interestingly, along with the advances in high-throughput sequencing and bioinformatics, a novel kind of RNA, named lncRNA, was recently discovered. LncRNAs have a sequence length of 200 bp or longer and have no protein-coding abilities, and they function as sponges for miRNAs or their parent/target genes (20). Meanwhile, within the SRSF1 intronic region, UCR-419 (named lnc-KASRT) fits the definition of a lncRNA and regulates KLF6 (a common tumor regulator) alternative splicing by sponging the SRSF1 gene. The role of lnc-KASRT and KLF6 alternative splicing in osteosarcoma development and progression is unclear. Therefore, our current study found that lnc-KASRT promoted osteosarcoma cell viability and mobility; moreover, lnc-KASRT regulated KLF6 alternative splicing to induce KLF6-SV1 while repressing KLF6-WT and regulating P21 and CCND1 expression in osteosarcoma. The possible explanations for these effects were as follows: (1) lnc-KASRT could regulate multiple oncogene/anti-oncogene alternative splicing events, including KLF6, to promote osteosarcoma cell malignant behaviors. (2) lnc-KASRT could directly sponge the SRSF1 gene itself to reduce SRSF1 expression, thus reversing KLF6 alternative splicing and reducing KLF6-WT while enhancing KLF6-SV1. (3) lnc-KASRT could regulate P21 and CCND1 upstream or directly regulate P21 and CCND1 *via* its alternative splicing ability.

KLF6, a common anti-oncogene, represses cell proliferation, migration, evasion, and epithelial-mesenchymal transition (EMT) and facilitates cell apoptosis and chemoradiotherapy sensitivity by regulating multiple pathways, such as the hedgehog, p53 apoptotic, EGFR, and p21/CCND1 pathways (31–34). In addition, it is fascinating that when the alternative splicing of KLF6 is disrupted or modified, KLF6 encodes KLF6-SV1 instead of KLF6-WT, which makes it act as an oncogene (19). Accumulating studies have reported that KLF6-SV1 promotes tumor development and progression in various ways, such as by regulating the NOXA and TWIST genes and the PI3K/AKT pathway (14–17). Considering the effect of lnc-KASRT on SRSF1 and KLF6 alternative splicing, we further explored the role of KLF6-SV1 in osteosarcoma malignant behaviors and whether it would affect the function of lnc-KASRT. Then, we found that KLF6-SV1 knockdown inhibited cell proliferation, migration, and invasion, promoted cell apoptosis, and regulated the P21/CCND1 pathway in osteosarcoma, which was in line with previous studies regarding the effect of KLF6-SV1 in other cancers. More importantly, we further discovered that KLF6-SV1 attenuated the effect of lnc-KASRT on regulating osteosarcoma cell functions and the P21/CCND1 pathway. These results indicated that lnc-KASRT indeed regulated osteosarcoma by modifying KLF6 alternative splicing.

Furthermore, to validate the above mentioned *in vitro* findings, we also performed *in vivo* experiments and found that lnc-KASRT promoted osteosarcoma growth and invasive markers while reducing tumor necrosis and apoptosis. Moreover, lnc-KASRT negatively regulated SRSF1 and KLF6-WT expression and positively modified KLF6-SV1 expression. These results also validated the notion that lnc-KASRT facilitated osteosarcoma progression by regulating KLF6 alternative splicing.

**Figure 11:**
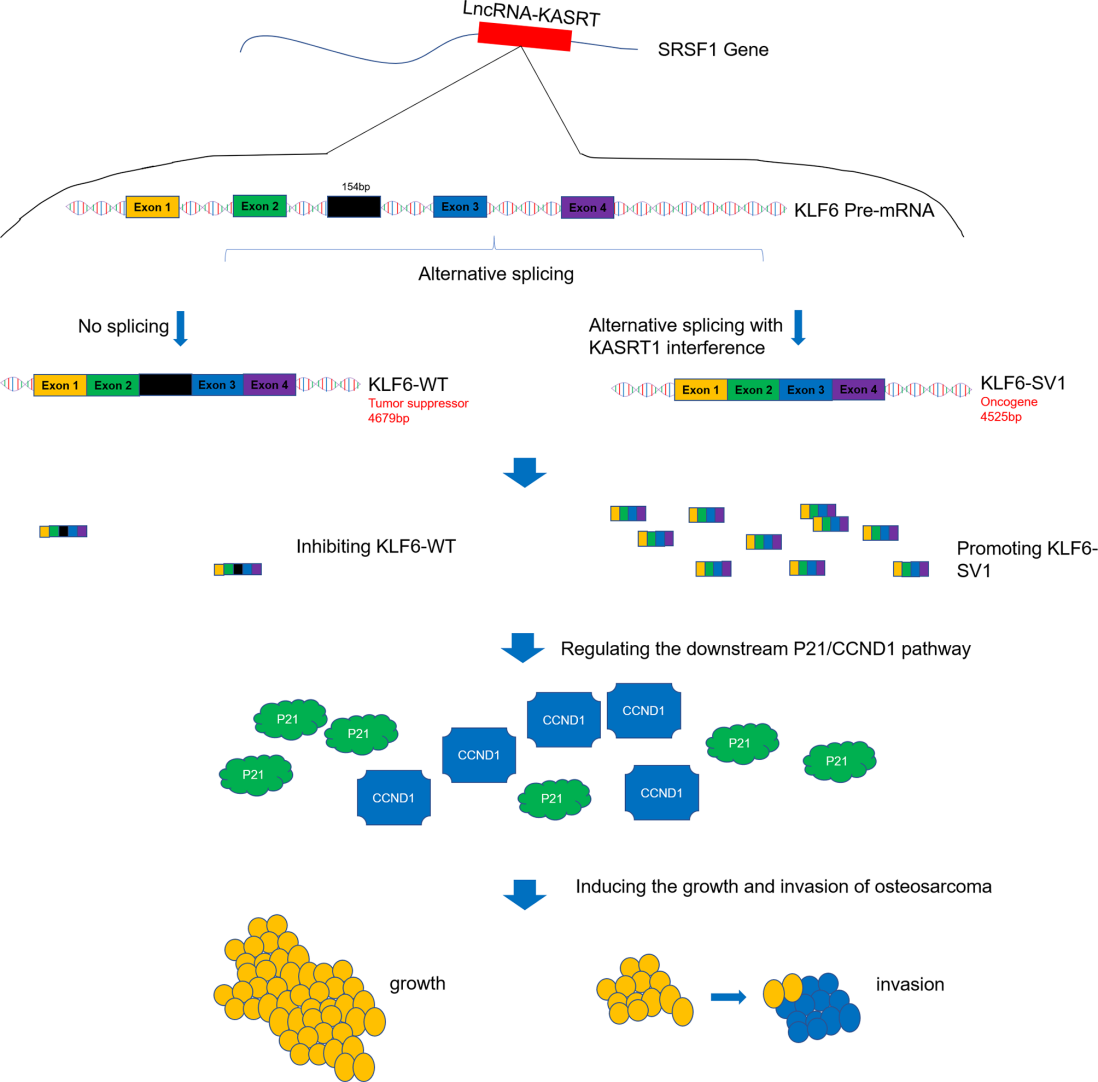
Visual summary of the main findings. Lnc-KASRT is located on SRSF1, interacts with SRSF1, and then regulates alternative splicing of KLF6 to promote KLF6-SV1 coding while inhibiting KLF6-WT coding, subsequently modifying the P21/CCND1 pathway, finally leading to increased growth and invasion of osteosarcoma.

To make the key findings of this study easy to interpret, a graphical figure illustrating the related mechanism was created (**Figure 11**). This visual summary shows that lnc-KASRT located on SRSF1, interacts with SRSF1, then regulates alternative splicing of KLF6 to promote KLF6-SV1 coding while inhibiting KLF6-WT coding, and subsequently modifies P21/CCND1 pathway, finally leading to increased growth and invasion of osteosarcoma.

There were some limitations in the current study. First, clinical human samples were not used for lnc-KASRT, SRSF1, KLF6-SV1, or KLF6-WT expression detection due to the limited number of patients. Second, the viability and mobility capacity were measured in this study; however, since our preliminary experiments observed that lnc-KASRT affects chemotherapy sensitivity little in U-2OS cells, we did not further explore the deep engagement of lnc-KASRT in treatment resistance, which could be considered in the future. Third, KLF6 was not only reported to be a tumor regulator but also an immune/inflammation trigger; therefore, the involvement of lnc-KASRT and KLF6 in the tumor immune microenvironment is another hotspot for further research. Fourth, *in silico* study to assess the application of lnc-KASRT as a target in osteosarcoma treatment was needed in the future.

In conclusion, lnc-KASRT serves as a potential treatment target by regulating SRSF1-related KLF6 alternative splicing and the P21/CCND1 pathway in osteosarcoma.

## Data Availability Statement

The original contributions presented in the study are included in the article/**Supplementary Material**. Further inquiries can be directed to the corresponding authors.

## Ethics Statement

All animal experiments were approved by the Institutional Animal Care and Use Committees (IACUC) of our institution and were performed in accordance with institutional guideline and ethical standard.

## Author Contributions

YC and XZ conceived and designed the study. KC, CL, and SH collected and analyzed the data. KC and CL prepared the figures and tables. KC, CL, and SH wrote the manuscript. YC and XZ edited the manuscript. All authors revised the manuscript and read and approved the submitted version.

## Funding

This study was supported by National Natural Science Foundation of China (No. 81602357) and Shanghai Health and Family Planning Commission Science Foundation (No.201740213).

## Conflict of Interest

The authors declare that the research was conducted in the absence of any commercial or financial relationships that could be construed as a potential conflict of interest.

## Publisher’s Note

All claims expressed in this article are solely those of the authors and do not necessarily represent those of their affiliated organizations, or those of the publisher, the editors and the reviewers. Any product that may be evaluated in this article, or claim that may be made by its manufacturer, is not guaranteed or endorsed by the publisher.

## Supplementary Material

Supplementary Figure 1 Chemosensitivity. Chemosensitivity of U-2OS cells to cisplatin **(A)**, methotrexate **(B)**, and doxorubicin **(C)**.
