# Supplementary figures and images for: LncRNA KASRT Serves as a Potential Treatment Target by Regulating SRSF1-Related KLF6 Alternative Splicing and the P21/CCND1 Pathway in Osteosarcoma: An In Vitro and In Vivo Study
Source: Front Oncol. 2021 Sep 9;11:700963. doi: 10.3389/fonc.2021.700963 (PMC8458968; doi:10.3389/fonc.2021.700963)

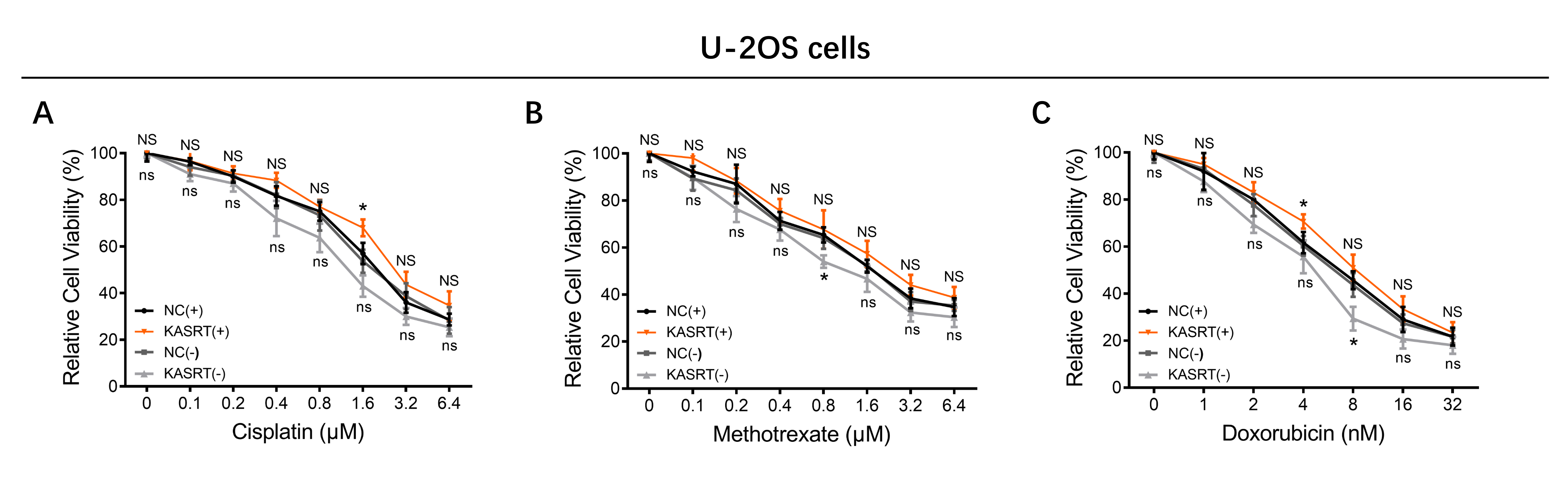

Supplement: Supplementary Figure 1 — Chemosensitivity. Chemosensitivity of U-2OS cells to cisplatin (A), methotrexate (B), and doxorubicin (C). [file Image_1.tif]

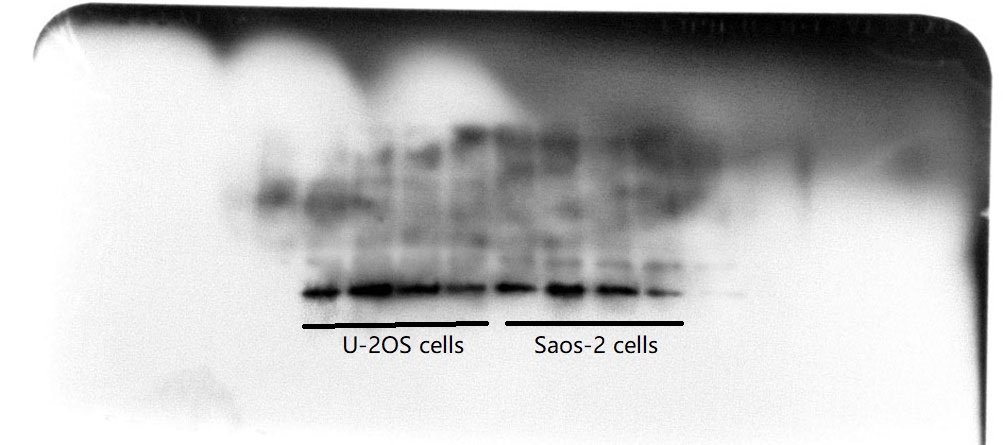

Supplement: Supplementary file 2 [file DataSheet_1.zip › Supplementary file_Western blot original images/Fig 2/Bcl-2.jpg]

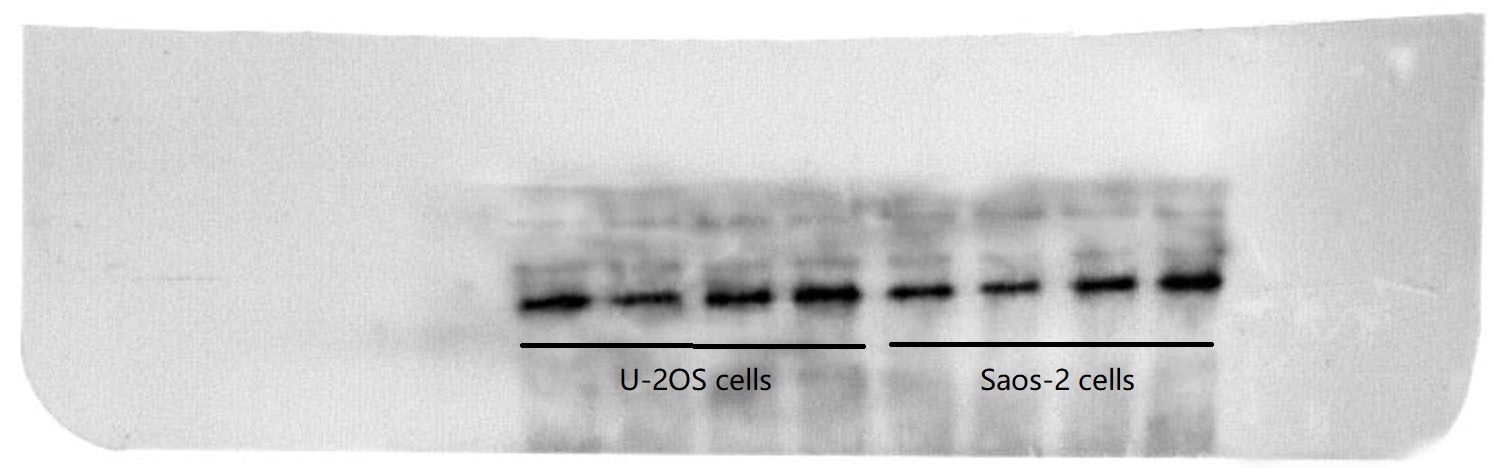

Supplement: Supplementary file 2 [file DataSheet_1.zip › Supplementary file_Western blot original images/Fig 2/C-Caspase3.jpg]

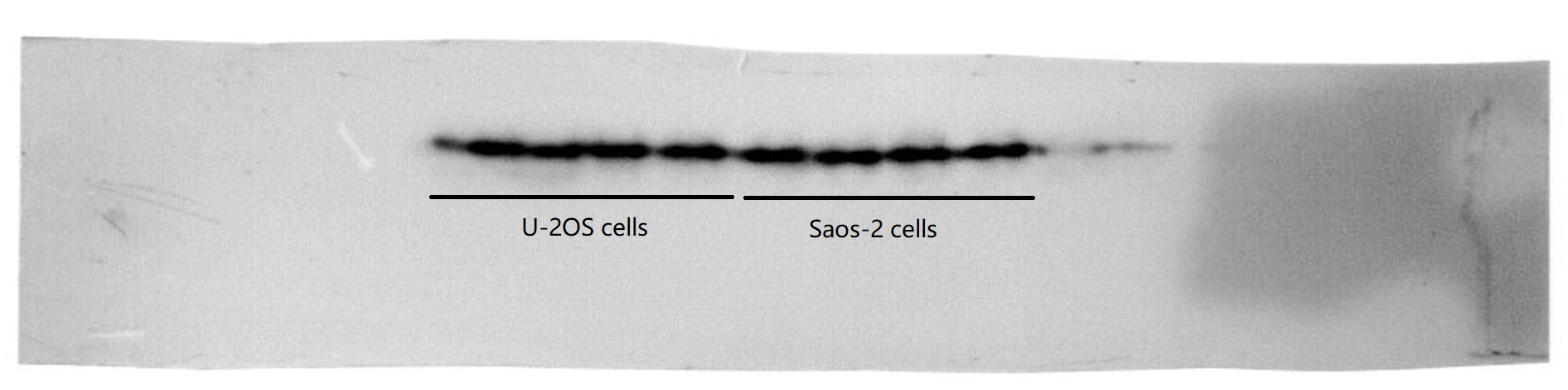

Supplement: Supplementary file 2 [file DataSheet_1.zip › Supplementary file_Western blot original images/Fig 2/Caspase3.jpg]

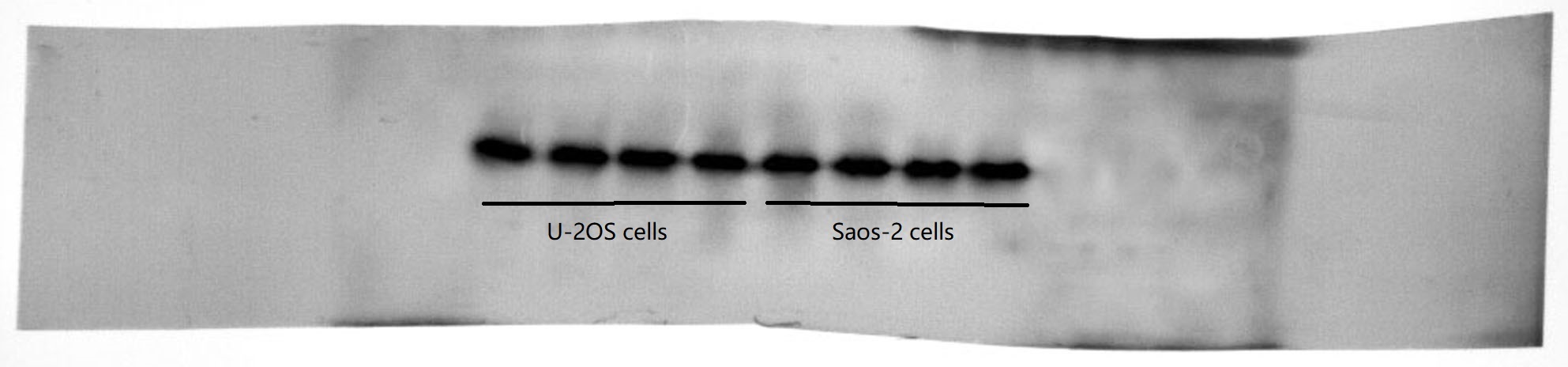

Supplement: Supplementary file 2 [file DataSheet_1.zip › Supplementary file_Western blot original images/Fig 2/GAPDH.jpg]

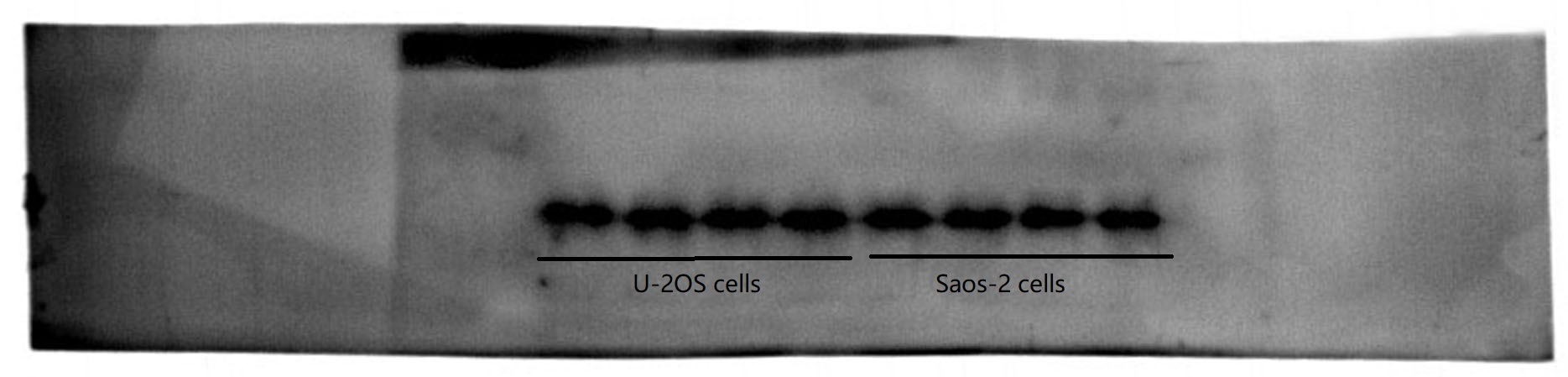

Supplement: Supplementary file 2 [file DataSheet_1.zip › Supplementary file_Western blot original images/Fig 4/GAPDH.jpg]

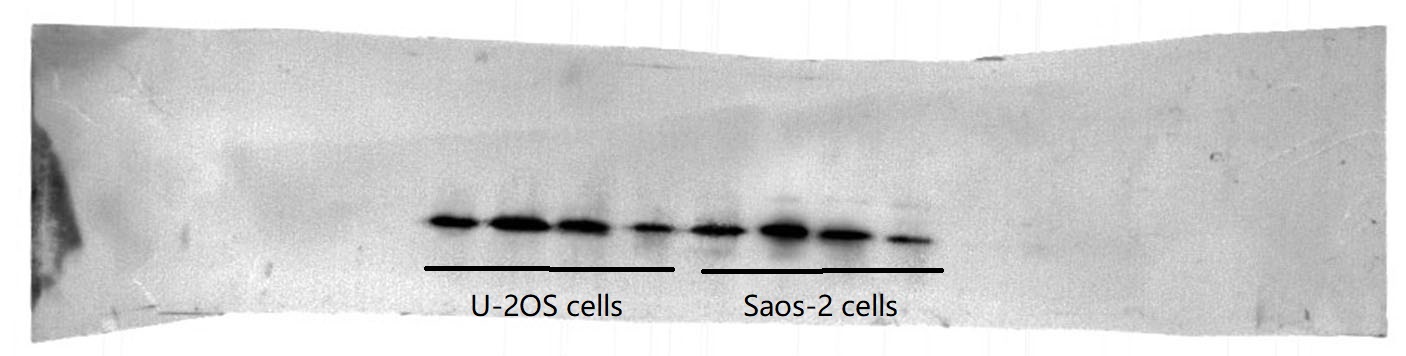

Supplement: Supplementary file 2 [file DataSheet_1.zip › Supplementary file_Western blot original images/Fig 4/KLF6-SV1.jpg]

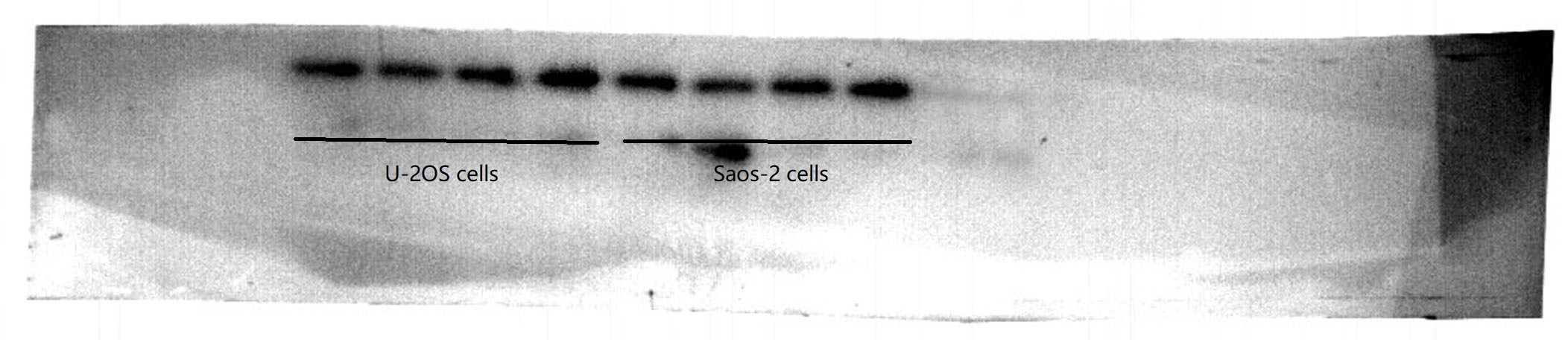

Supplement: Supplementary file 2 [file DataSheet_1.zip › Supplementary file_Western blot original images/Fig 4/KLF6-WT.jpg]

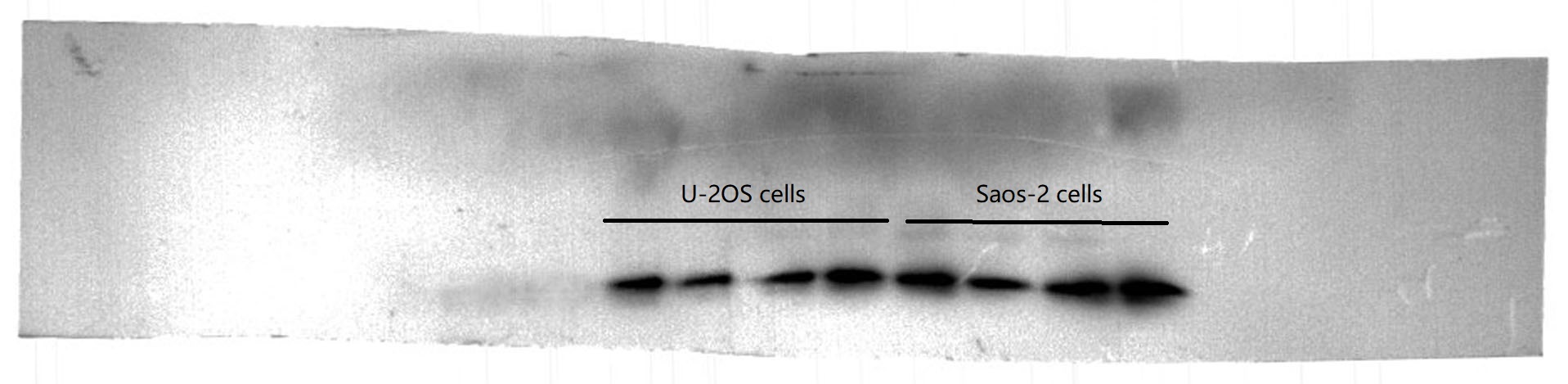

Supplement: Supplementary file 2 [file DataSheet_1.zip › Supplementary file_Western blot original images/Fig 4/SRSF1.jpg]

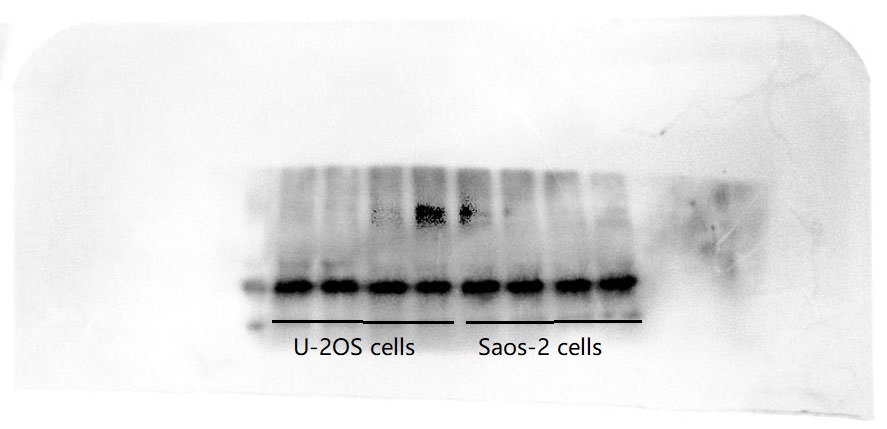

Supplement: Supplementary file 2 [file DataSheet_1.zip › Supplementary file_Western blot original images/Fig 5/GAPDH.jpg]

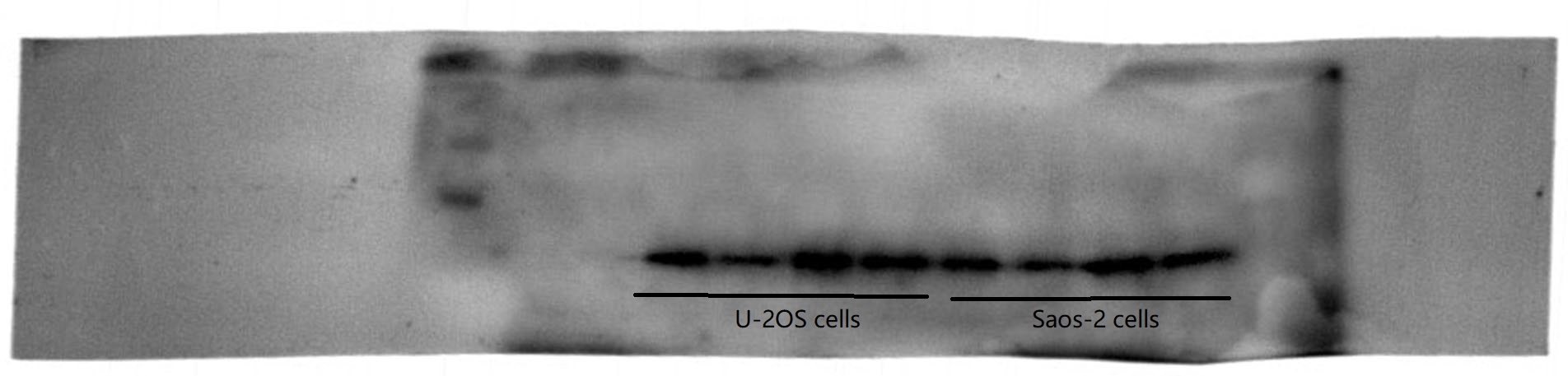

Supplement: Supplementary file 2 [file DataSheet_1.zip › Supplementary file_Western blot original images/Fig 5/KLF6-SV1.jpg]

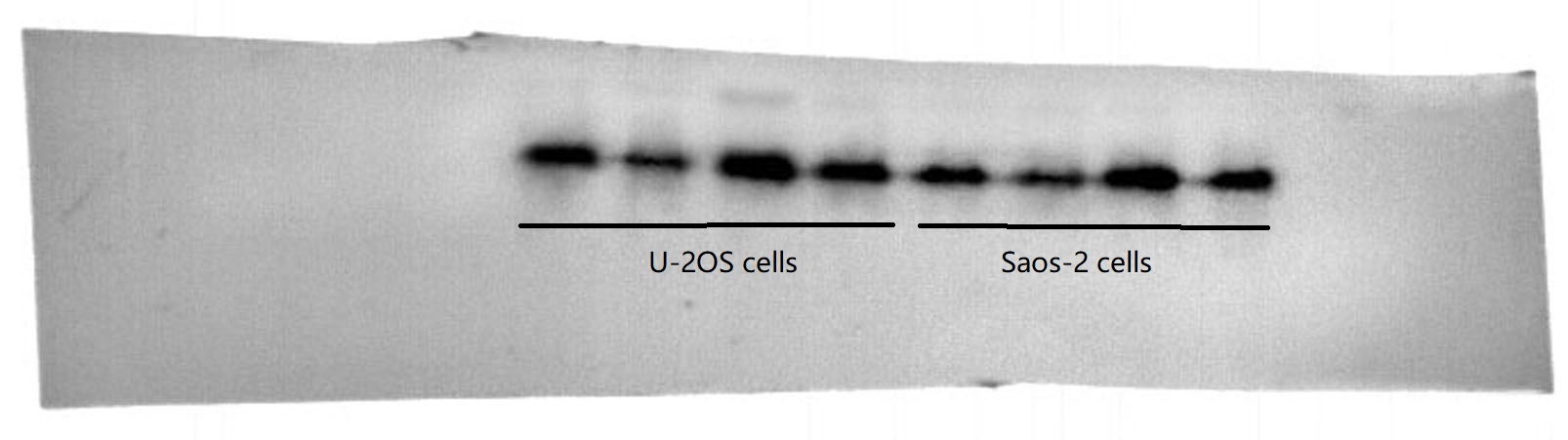

Supplement: Supplementary file 2 [file DataSheet_1.zip › Supplementary file_Western blot original images/Fig 6/Bcl-2.jpg]

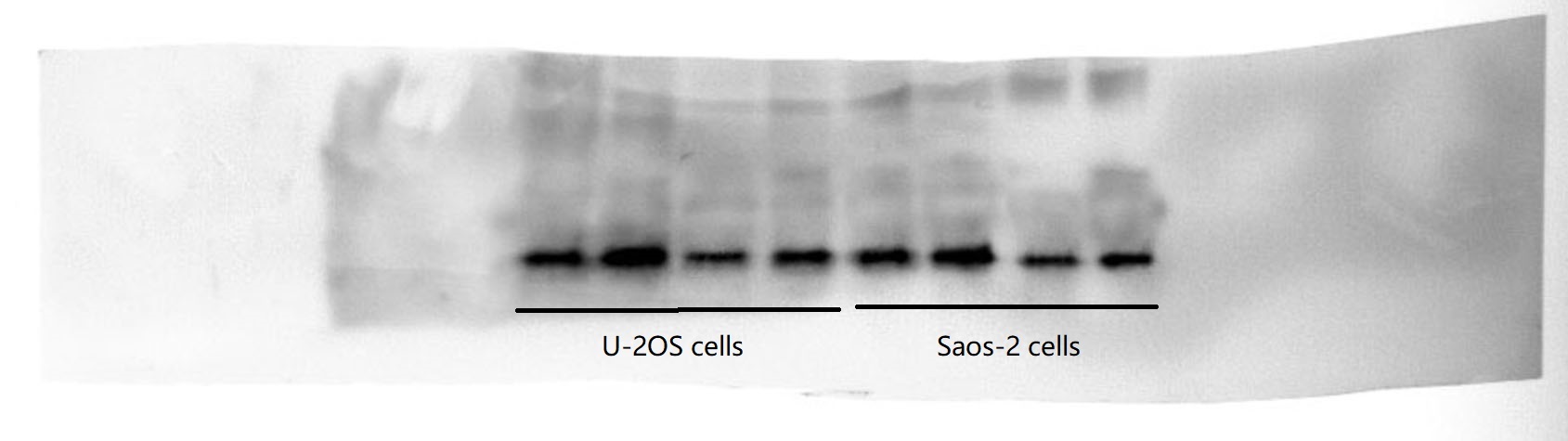

Supplement: Supplementary file 2 [file DataSheet_1.zip › Supplementary file_Western blot original images/Fig 6/C-Caspase3.jpg]

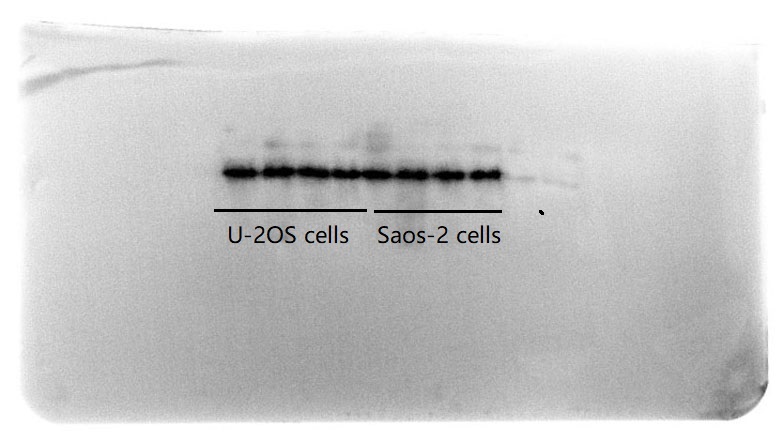

Supplement: Supplementary file 2 [file DataSheet_1.zip › Supplementary file_Western blot original images/Fig 6/Caspase 3.jpg]

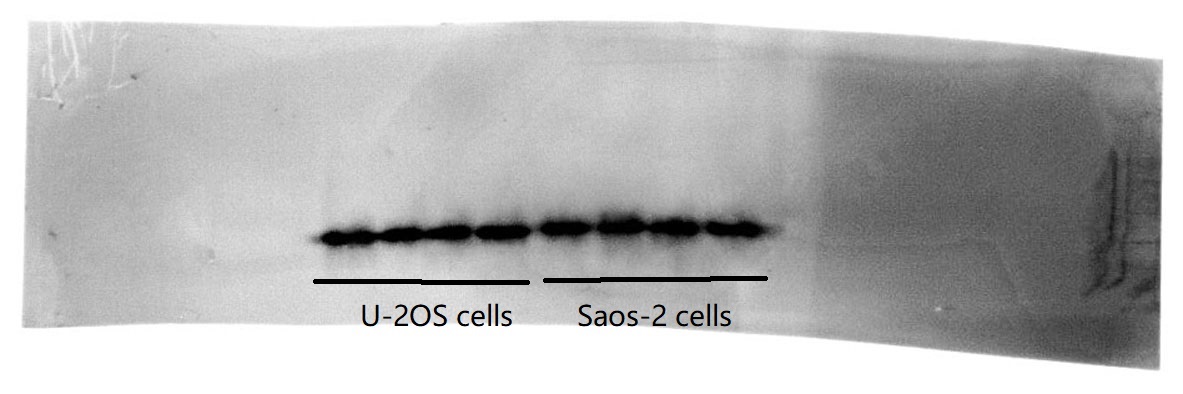

Supplement: Supplementary file 2 [file DataSheet_1.zip › Supplementary file_Western blot original images/Fig 6/GAPDH.jpg]

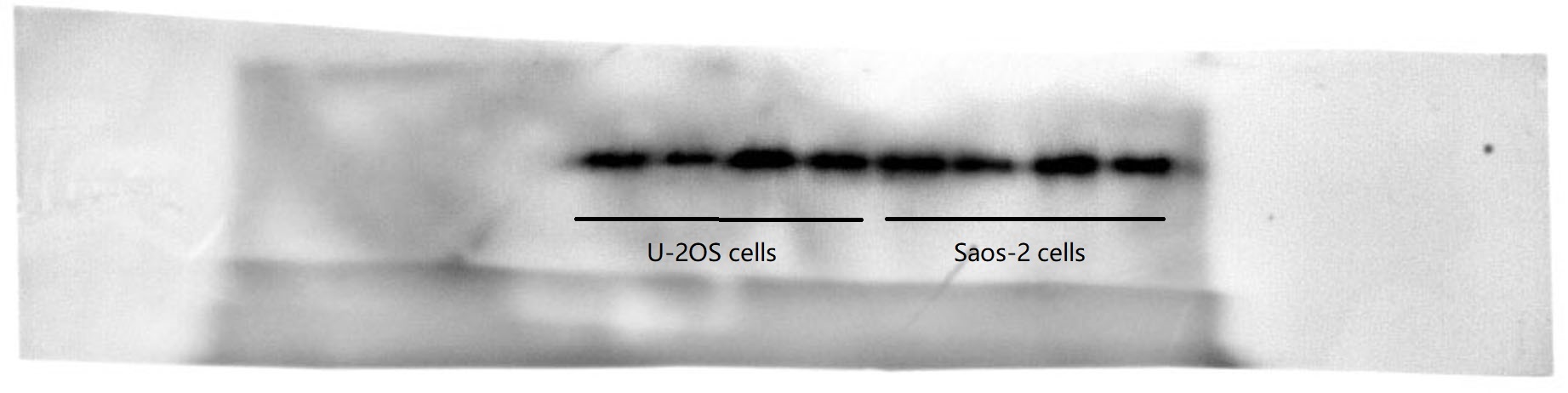

Supplement: Supplementary file 2 [file DataSheet_1.zip › Supplementary file_Western blot original images/Fig 8/CCND1.jpg]

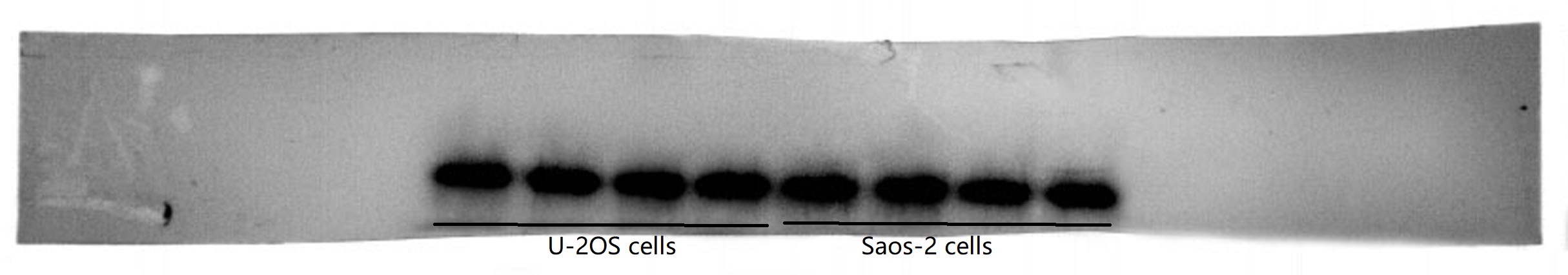

Supplement: Supplementary file 2 [file DataSheet_1.zip › Supplementary file_Western blot original images/Fig 8/GAPDH.jpg]

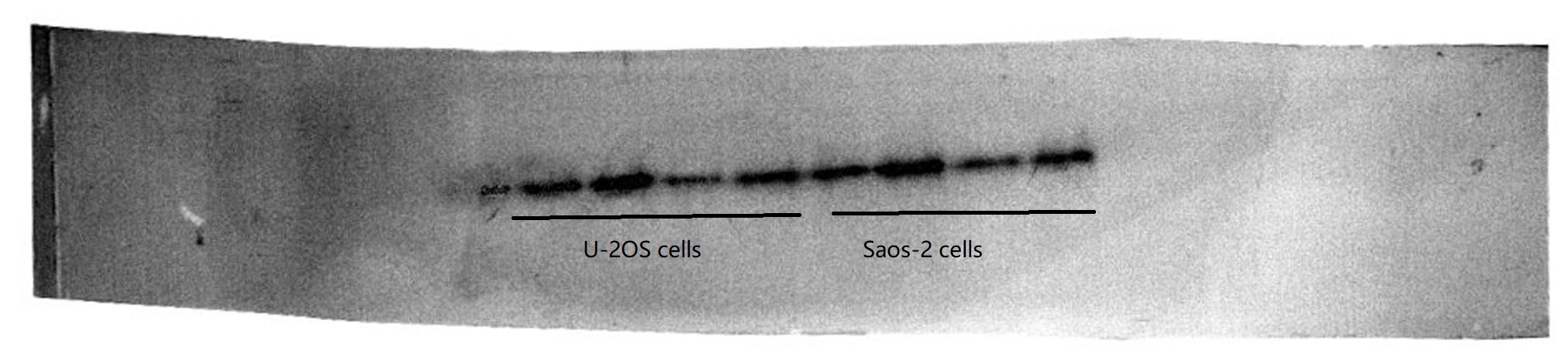

Supplement: Supplementary file 2 [file DataSheet_1.zip › Supplementary file_Western blot original images/Fig 8/P21.jpg]
