# Supplementary material for: LncRNA KASRT Serves as a Potential Treatment Target by Regulating SRSF1-Related KLF6 Alternative Splicing and the P21/CCND1 Pathway in Osteosarcoma: An In Vitro and In Vivo Study
Source: Front Oncol. 2021 Sep 9;11:700963. doi: 10.3389/fonc.2021.700963 (PMC8458968; doi:10.3389/fonc.2021.700963)
Supplement: Supplementary file 3 [file Table_1.docx]

**Supplementary table 1.** Primers sequences

| Gene | Forward Primer (5’-3’) | Reverse Primer (5’-3’) |
| --- | --- | --- |
| Lnc-KASRT | AGGTGGCTGCTGGAGAGAAA | GACTGAGGTGGCTTACTGATGG |
| SRSF1 | ACCTCCAGACATCCGAACCAA | CGAACTCAACGAAGGCGAAGG |
| KLF-6-SV1 | CCTCGCCAGGGAAGGAGAA | CGGTGTGCTTTCGGAAGTG |
| KLF-6-WT | CGGACGCACACAGGAGAAAA | CGGTGTGCTTTCGGAAGTG |
| P21 | CGTCCTCGGATTCTCTGCTCTC | GCTGCGTAGTTGTGCTGATGT |
| CCND1 | CCAGAGGCGGATGAGAACAAG | GCGGTAGCAGGAGAGGAAGT |
| MMP-1 | AGATGTGGAGTGCCTGATGTG | CTTGGTGAATGTCAGAGGTGTGA |
| MMP-9 | GCACCACCACAACATCACCTA | GGACCACAACTCGTCATCGT |
| GAPDH | GACCACAGTCCATGCCATCAC | ACGCCTGCTTCACCACCTT |
