# Supplementary material for: LncRNA KASRT Serves as a Potential Treatment Target by Regulating SRSF1-Related KLF6 Alternative Splicing and the P21/CCND1 Pathway in Osteosarcoma: An In Vitro and In Vivo Study
Source: Front Oncol. 2021 Sep 9;11:700963. doi: 10.3389/fonc.2021.700963 (PMC8458968; doi:10.3389/fonc.2021.700963)
Supplement: Supplementary file 4 [file Table_2.docx]

**Supplementary table 2.** Antibodies

| Antibody | Company | Dilution (western blot/IHC)) |
| --- | --- | --- |
| **Primary Antibody** |  |  |
| Caspase-3 Rabbit mAb | CST (USA) | 1:1000/- |
| Cleaved Caspase-3 Rabbit mAb | CST (USA) | 1:1000/- |
| Bcl-2 Rabbit mAb | CST (USA) | 1:1000/- |
| SRSF1 Rabbit mAb | Abcam (UK) | 1:5000/1:100 |
| KLF-6-SV1 Rabbit pAb | Fab Gennix (USA) | 1:500/1:100 |
| KLF-6 mouse mAb | Santa Cruz (USA) | 1:2000/1:200 |
| P21 Rabbit mAb | CST (USA) | 1:1000/1:50 |
| Cyclin D1 (CCND1) Rabbit mAb | CST (USA) | 1:1000/1:500 |
| MMP-1 Rabbit mAb | Abcam (UK) | 1:1000/1:200 |
| MMP-9 Rabbit mAb | Abcam (UK) | 1:1000/1:1000 |
| GAPDH Rabbit mAb | CST (USA) | 1:1000/1:800 |
| **Secondary Antibody** |  |  |
| Goat Anti-mouse IgG-HRP | Santa Cruz (USA) | 1:5000/1:1000 |
| Goat Anti-Rabbit IgG H&L (HRP) | Abcam (USA) | 1:10000/1:1000 |
